# Promoting early childhood oral health and preventing early childhood caries on Instagram

**DOI:** 10.3389/froh.2022.1062421

**Published:** 2023-01-05

**Authors:** Victor H. K. Lee, Grace Kyoon-Achan, Josh Levesque, Suhird Ghotra, Ralph Hu, Robert J. Schroth

**Affiliations:** ^1^ Department of Preventive Dental Science, Dr. Gerald Niznick College of Dentistry, Rady Faculty of Health Sciences, University of Manitoba, Winnipeg, MB, Canada; ^2^ Children’s Hospital Research Institute of Manitoba, Winnipeg, MB, Canada; ^3^ Department of Pediatrics and Child Health, Max Rady College of Medicine, Rady Faculty of Health Sciences, University of Manitoba, Winnipeg, MB, Canada; ^4^ Section of Pediatric Dentistry, Winnipeg Regional Health Authority, Winnipeg, MB, Canada; ^5^ Shared Health Inc., Winnipeg, MB, Canada

**Keywords:** oral health, health promotion, child health, chronic disease, public health, social media

## Abstract

**Introduction:**

Early childhood caries (ECC) is prevalent worldwide. Oral health promotion effectively utilizes key messages to educate parents/caregivers and the public on how to prevent ECC. Instagram is one of the biggest social media platforms, and could be used to promote early childhood oral health. The purpose of this study was to determine if and how young children's oral health is promoted and supported on Instagram.

**Methods:**

This study used inductive content analysis to categorize, quantify, and interpret pictorial and textual data derived from Instagram posts containing the most commonly used ECC-related hashtags in their captions (determined by an extensive search through Instagram's search bar).

**Results:**

A total of 1,071 images and 3,228 comments were analyzed based on 13 hashtags. The most common types of images were those of people (57.5%) and graphics/memes (37.8%). Most people were older children (32.5%) or adults (20.3%), and were White (19.6%) or Asian (18.5%). A majority of images had people posing (79.1%) in dental clinics (81.3%). Most graphics/memes were instructional/informational (76.3%). A total of 173 posts had substantial discussions that were positive/constructive in nature. The majority of discussions had at least one comment providing advice, tips, or explanations (79.8%), or had users requesting further information (73.4%).

**Conclusion:**

As more people engage with social media, health professionals should consider the potential for Instagram as a tool to promote early childhood oral health and to prevent ECC. Our study shows that many different users are providing and consuming content related to ECC. Targeted messaging, monitoring of content, and professional guidance could be beneficial to those seeking oral health information on this platform.

## Introduction

Early childhood caries (ECC), or caries in the primary dentition of children under six years of age, is prevalent worldwide. Behavioural risk factors include poor oral hygiene (e.g., not brushing with fluoridated toothpaste and not visiting a dentist regularly) and poor nutrition and feeding habits (e.g., frequent exposure to dietary fermentable carbohydrates that cariogenic bacteria use for growth) (1, 2). Early childhood oral health sets the foundation for good dental health throughout childhood and adolescence. Those who experience ECC at a young age have an increased risk of developing future caries in both the primary and permanent dentitions (3–5). Considerable efforts should be devoted to the prevention of ECC. Preventative measures are preferred and necessary to reduce the need for more serious treatments.

Oral health promotion is an effective method that utilizes key messages to educate parents, caregivers, and the public on how to prevent tooth decay in young children (6, 7). Oral health promotion campaigns can be implemented to promote oral health and hygiene by increasing awareness of its importance, and by supporting parents and caregivers of young children through social media. Social media usage on platforms such as Instagram, Facebook, Twitter, and YouTube have become more common with increased online activities globally. Nearly four billion people all over the world consume a wide range of information on these platforms every day (8). The increased use and advanced technology of social media provides greater opportunities for inquiry, learning, and insight. However, not all health-related information found on social media is of high quality, and there is the risk of unprofessional and detrimental content reaching the masses (9). One study examined YouTube as a patient source for information on ECC and concluded that it is not reliable, and should not be used without guidance to educate parents and caregivers (10).

Research describing the use of other social media platforms as a tool for oral health promotion is scarce (11–13). As one of the major platforms today, there is limited information on the role of Instagram in oral health promotion and education. Instagram is a photo and video sharing application that has over one billion active users worldwide (14). Effective communication through Instagram is possible through user posts of images and videos, captions and comments on posts, and direct messaging. Discovery of common interests, such as oral health topics, are facilitated by the application's algorithm (based on the user's activity) and by hashtags (#). Hashtags contain key words or terms that users attach to the captions or comments of posts. It allows the users to see what other images and videos are associated with a particular term. The immense, convenient, and accessible nature of Instagram may be conducive to effective oral health promotion. Large amounts of information can be distributed and consumed by those using the application. The purpose of this study was to determine if and how early childhood oral health is promoted and supported on Instagram.

## Methods

This study used inductive content analysis to help categorize and interpret pictorial and textual data derived from Instagram for the purpose of understanding and describing early childhood oral health concepts (15, 16). The most commonly used hashtags related to ECC, early childhood oral health, and oral health promotion were identified by using Instagram's own search bar to see the number of posts containing a particular term in its caption. An extensive search was done on current and previous terms used in captions about ECC, oral health, and oral health promotion for images posted on or before 31 July 2020. The list of terms searched is provided as supplementary material. Acronyms were not considered in our search due to the broad range of images associated with such terms.

To keep the study focused, only caries-related hashtags were selected for further analysis. The chosen terms are as follows: #babybottlecaries, #babybottlesyndrome, #babybottletoothdecay, #circularcaries, #earlychildhoodcaries, #earlychildhoodoralhealth, #earlychildhoodtoothdecay, #nursingbottlecaries, #nursingbottlesyndrome, #nursingcaries, #preventearlychildhoodcaries, #preventearlychildhoodtoothdecay, and #rampantcaries. Images may not have been unique or exclusive to a single term, as users may have added additional hashtags to the caption of a single post. Reposted images and duplicates of images were retained for analysis. The study team only examined posts that were visible on users' accounts and did not consider archived or deleted posts. We did not seek ethics approval/consent from users, as we examined information that was publicly available and non-identifying.

Trained research staff completed both the standardized collection and analysis of data. We used the inductive content analysis approach, as no previous studies exist as a guide for Instagram-based oral health promotion (15). We identified and quantified the frequency of themes in images, captions, and comments collected for the study (17). Two researchers (V.L. and J.L.) collected and utilized ideas or concepts that emerged from reviews of data to shape coding frames. For each Instagram post, we used inductive coding methods to analyze images first, and then we moved on to associated captions and comments. Simple descriptive analysis was also used. A coding guide is provided as supplementary material.

For images, the general type of image was recorded first. Specific details about people, activities, objects, graphics, and text depicted in images were added later. Captions were coded based on their purpose (e.g., praising or agreeing, critiquing, thanking, providing advice or information, promoting, reflecting, asking questions, etc.). For comments, posts with six or more comments were considered to have a “substantial discussion”, and were included for analysis (16). These comments were also coded based on their purpose. Non-English captions and comments were translated and included in our analysis. Captions and comments with generalized hashtags were coded as “other”. Image coding and the resulting dataset were constantly reviewed to ensure theme consistency, reliability, and trustworthiness (18). Data were analyzed using Number Cruncher Statistical Software (Kaysville, Utah). Descriptive statistics were reported.

## Results

The dataset consisted of 1,071 images and 3,228 comments (median: one comment per image (Table 1). Of the 13 selected hashtags, #earlychildhoodcaries had the most posts, with 559 images and 1,928 comments. One image under #earlychildhoodcaries had a discussion consisting of 111 comments. The hashtag #circularcaries had the least amount of posts, with only one image and no comments.

**Table 1:** Images and associated comments posted on Instagram before or on 31 July 2020 using the top oral health-related hashtags.

Hashtag	Number of images	Number of comments	Comments per image		
			Mean	Median	Max
#circularcaries	1	0	0.0	0	0
#preventearlychildhoodcaries	3	0	0.0	0	0
#nursingbottlesyndrome	3	1	0.3	0	1
#preventearlychildhoodtoothdecay	6	3	0.5	0	2
#earlychildhoodtoothdecay	7	12	1.7	0	11
#earlychildhoodoralhealth	7	2	0.3	0	1
#nursingcaries	34	111	3.3	2	27
#babybottlesyndrome	51	100	2.0	1	14
#babybottlecaries	61	221	3.6	0	78
#rampantcaries	78	152	1.9	0	21
#nursingbottlecaries	99	470	4.7	0	81
#babybottletoothdecay	162	228	1.4	0	94
#earlychildhoodcaries	559	1,928	3.4	1	111
Total	1,071	3,228	3.0	1	111

Our analysis of images is presented in Table 2. The most common image type was those of people (*n* = 616/1,071, 57.5%). These images often featured an older child (*n* = 200/616, 32.5%), an infant (*n* = 79/616, 12.8%), or adults (*n* = 125/616, 20.3%). Of the images focusing on people, 29.9% were female, and 25.8% were male (includes images with both males and females). More than half (53.4%) of images did not specify the individual's sex or gender. Most images contained people that were White (*n* = 121/616, 19.6%) or Asian (*n* = 114/616, 18.5%). There was little representation of Black (*n* = 16/616, 2.6%), Indigenous (*n* = 2/616, 0.3%), or other individuals of different backgrounds.

**Table 2:** Analysis of images from selected oral health-related hashtags on Instagram posted before or on 31 July 2020.

Image type, subtype categories, or features	No. of images	% of total images (*N* = 1,071)	% of defined subset
**People**		616	57.5	
Age	Adults in image	34	3.2	5.5
	Children in image	491	45.8	79.7
	Newborns	9	0.8	1.8
	Infants	70	6.5	14.3
	Toddlers	35	3.3	7.1
	Older children	133	12.4	27.1
	Age unclear/unspecified	244	22.8	49.7
	Images with both adults and children in image	91	8.5	14.8
	Adults with newborns	9	0.8	9.3
	Adults with infants	9	0.8	9.3
	Adults with toddlers	6	0.5	6.6
	Adults with older children	67	6.3	73.6
Sex	Male	103	9.6	16.7
	Female	128	12.0	20.8
	Unspecified	329	30.7	53.4
	Both males and females	56	5.2	9.1
Background	White	121	11.3	19.6
	Black	16	1.5	2.6
	Asian	114	10.6	18.5
	Indigenous	2	0.2	0.3
	Other (includes unspecified)	363	33.9	58.9
Activities	Brushing teeth	11	1.0	1.8
	Child brushing alone	7	0.7	63.6
	Child brushing with adult supervision/help	4	0.4	36.4
	Eating	1	0.1	0.2
	Drinking	60	5.6	9.7
	Bottle-feeding	55	5.1	91.7
	Using sippy cup	4	0.4	6.7
	Other	1	0.1	1.7
	Providing/receiving dental care	51	4.8	8.3
	Posing	487	45.5	79.1
	Posing in dental clinic	396	37.0	81.3
	Posing elsewhere	86	8.0	17.7
	Other	1	0.1	0.2
**Food and/or drink**		6	0.6	0
Objects		57	5.3	0
	Related to oral health	38	3.5	66.7
	Toothbrushes	4	0.4	10.5
	Toothpaste	1	0.1	2.6
	Dental floss	11	1.0	28.9
	Bottle or sippy cup	2	0.2	5.3
	Other	20	1.9	52.6
	Not related to oral health	19	1.8	33.3
Graphics and memes		405	37.8	0
	Oral health reflection and/or opinion	7	0.7	1.7
	Screenshots	23	2.1	5.7
	Instructional/informational	309	28.9	76.3
	Promotional	65	6.1	16.0
	Other	1	0.1	0.2
Images with text		620	57.9	0
	Text only	2	0.2	0
	Motivational quotes	4	0.4	0.6
	General oral health information	6	0.6	1.0
	Instructional text	370	34.5	59.7
	Promotional text	238	22.2	38.4
	Brand names and/or logos	221	20.6	92.9
	Social media promotion	3	0.3	1.3
	Promotions for presentations	10	0.9	4.2
	Other promotional text	4	0.4	1.7
	Other	7	0.7	1.1
Other		7	0.7	0

Most images of people had them posing for the camera (*n* = 487/616, 79.1%). A majority of individuals were posing in a dental clinic (*n* = 396/487, 81.3%). This includes clinical images of children's teeth (with and without dental caries). Only 11 images of people had them brushing their teeth (1.8%). No images featured people flossing or using mouthwash. About 10% of people in images were shown drinking (*n* = 60/616). Most posts had children drinking from a bottle (*n* = 55/60, 91.7%). Few images showed only foods or drinks (*n* = 6/1,071, 0.6%). Only a small amount of images showed people providing or receiving dental care (*n* = 51/616, 8.3%). These pictures included wide shots of dental care providers with their patients, and close-up images of specific treatments.

Only 5.3% of images featured objects (*n* = 57/1,071). Of these images, most objects were related to oral health (*n* = 38/57, 66.7%). The main objects related to oral health included toothbrushes, toothpaste, dental floss, bottles, or sippy cups. Other objects included dental equipment, holiday decorations, and toys.

More than a third of all images were of graphics or memes (*n* = 405/1,071, 37.8%). The majority of graphics and memes were informational and instructional (*n* = 309/405, 76.3%), or promotional (*n* = 65/405, 16%). Informational and instructional posts highlighted etiological factors of ECC and good and bad oral hygiene habits (e.g., brushing habits). Over half of all images contained text (*n* = 620/1,071, 57.9%). Of these images, the purpose of most of the text was instructional (*n* = 370/620, 59.7%) or promotional (*n* = 238/620, 38.4%) in nature. A vast majority of promotional text was found in the form of brand names and logos (*n* = 221/238, 92.9%). This included dentist or practice information (i.e., office/professional marketing for prospective patients). Very few images contained text only (*n* = 2/1,071, 0.2%).

A total of 173 posts had six comments or more, and were considered “substantial discussions”. Table 3 shows the most common image caption elements and comment discussion elements. A majority of captions provided general information on oral health and dental care (*n* = 765/1,071, 71.4%). Most captions also provided advice or tips without recommending a product (*n* = 385/1,071, 35.9%), and reflected on personal experiences relating to a child (*n* = 304/1,071, 28.4%).

**Table 3:** Analysis of captions and comments associated with images from selected oral health-related hashtags posted before or on 31 July 2020.

Image caption elements	No. (%) of total caption elements (*N* = 2,246)	% of captions (*N* = 1,071)
Praising, complementing, and/or agreeing with others	12 (0.5%)	12 (1.1%)
Disagreeing with/critiquing others	2 (0.1%)	2 (0.2%)
General complaining	2 (0.1%)	2 (0.2%)
Thanking	17 (0.8%)	17 (1.6%)
Recommending products	12 (0.5%)	12 (1.1%)
Giving advice/tips, but not recommending products	384 (17.1%)	384 (35.9%)
General information on oral health and dental care	765 (34.1%)	765 (71.4%)
Promotional captions	372 (16.6%)	372 (34.7%)
Reflecting on personal experiences not related to child	77 (3.4%)	77 (7.2%)
Reflecting on personal experiences related to child	304 (13.6%)	304 (28.4%)
Opinion/reflection on dental care/oral health practices and ideas	150 (6.7%)	150 (14.0%)
Expressing concern with cultural, societal, or financial components of oral health	5 (0.2%)	5 (0.5%)
Research-related caption	34 (1.5%)	34 (0.2%)
Asking a question (nonrhetorical)	56 (2.5%)	56 (5.2%)
Other	54 (2.4%)	54 (5.0%)
Content discussion elements	No. (%) of total discussion elements (*N *= 2,610)	% of discussions (*N* = 173)
Giving advice, tips, and/or explanations, but not recommending products	731 (28.0%)	138 (78.8%)
Asking a question and/or requesting further information	450 (17.2%)	127 (73.4%)
Praising, complementing, and/or agreeing with a caption/comment	445 (17.0%)	129 (74.6%)
Praising, complementing, and/or agreeing with another user, without regard for their original caption/comment	179 (6.9%)	49 (28.3%)
Disagreeing with, contesting, and/or critiquing a caption/comment	8 (0.3%)	5 (2.9%)
Disagreeing with, contesting, and/or critiquing another user, without regard for their original caption/comment.	3 (0.1%)	3 (1.7%)
Including another Instagram user	298 (11.4%)	95 (54.9%)
Thanking	256 (9.8%)	111 (64.2%)
Reflecting on personal experiences related to child	114 (4.4%)	47 (27.2%)
Reflecting on personal experiences not related to child	41 (1.6%)	24 (13.9%)
Reflecting positively on dental care/oral health practices and ideas	11 (0.4$)	5 (2.9%)
Reflecting negatively on dental care/oral health practices and ideas	8 (0.3%)	5 (2.9%)
Expressing concern with cultural, societal, or financial component of oral health	6 (0.2%)	6 (3.5%)
Recommending products	5 (0.2%)	2 (1.2%)
General complaining	4 (0.2%)	3 (1.7%)
Other	51 (2.0%)	35 (20.2%)

Overall, discussions on Instagram were very positive. The majority of discussions had at least one comment giving advice, tips, or explanations, without recommending products (*n* = 138/173, 79.8%); had questions or individuals requesting further information (*n* = 127/173, 73.4%); or had individuals praising, complementing, or agreeing with a caption or comment (*n* = 129/173, 74.6%). Very few discussions contained negative comments. These would include comments disagreeing with, contesting, or critiquing a caption or comment(s) (n = 3/173, 1.7%); reflecting negatively on dental care/oral health practices and ideas (*n* = 5/173, 2.9%); or any general complaining (*n* = 3/173, 1.7%).

## Discussion

Results suggest that early childhood oral health is being promoted and supported on Instagram through user posts and conversations. The abundance of images associated with #earlychildhoodcaries highlights the significance of the term as a common descriptor of dental caries in the primary dentition of children (19). Images widely covered the etiology and treatment of ECC through clinical images, and informational, instructional, and promotional graphics and memes. There was very little visual representation of behaviours or objects related to the prevention of ECC, however. Few images had people brushing their teeth or flossing, or had images of toothbrushes, toothpaste, and dental floss themselves. Information on prevention was mostly textual.

Image captions and comments reflected users' positive attitudes towards the use of Instagram as a source of information, and as a platform for discourse and support. Analysis of discussion elements showed that users were willing to share and seek out information about ECC through the application. The use of Instagram as a means of communication could provide oral health benefits for parents/caregivers and their children. Farnan et al. describes the use of supplemental electronic communication to communicate with patients and improve treatment adherence in those with chronic diseases (20). Over the last two years, our Healthy Smile Happy Child community outreach initiative has embraced the use of Instagram (@healthysmilehappychild) to reach families in Manitoba, Canada with early childhood oral health messaging, raise awareness of oral health events happening in communities, and to even promote research studies related to ECC. Some of the posts included in our analyses were from the Healthy Smile Happy Child Instagram account.

Research shows that individuals are interested in learning more about children's oral health and are seeking out information on the topic. One study evaluated the behaviour of Internet users from seven countries (Brazil, France, Italy, Mexico, Spain, United Kingdom, and United States) by looking at Google search trends (by volume) between January 2004 and December 2020. The authors discovered increasing levels of interest in ECC-related information, as it relates to definitions, risk factors, and preventive care (21). Overall search levels remained low, however, which could indicate a lack of awareness of ECC as a potential problem in the early stages of life.

As mentioned above, not all health-related information disseminated on social media is of high quality, and poor content can be shared. Aguirre et al. used the world's most popular search engines and various standards of measurement to assess the quality and readability of ECC-related web information in English, Portuguese, and Spanish (22). While web content could be easily read, it was of low quality irrespective of language or authorship. These findings highlight the importance of professional counseling to help parents or caregivers make the best decisions for their child's oral health (22). The credibility of information found on social media, along with its sources, should be questioned, as there is no guarantee on their quality. There is a lot of easily accessible information available for people to consume, and health care organizations, health care professionals, and the public all have the responsibility of ensuring its authenticity (23, 24).

Effective communication of health information should also understand and acknowledge the target audience. Diverse messaging can help address inequities that exist in media representation (25). Results showed that there were disparities in visual representations when it came to race, but not other characteristics. Most images were of White or Asian people, and there was little representation of Black, Indigenous, or other individuals. An American study examining racial and ethnic representation of people featured in hospitals' social media platforms (Facebook, Twitter, and YouTube) had similar findings. When compared with the demographics of hospitals' neighboring communities, Whites and Asians were over-represented in images and videos (25).

The descriptive nature of this study is a notable limitation. This study cannot and does not examine reactions to Instagram content beyond initial posts, captions, and comments. Online interactions may extend beyond these points of engagement through additional information-seeking behaviours, and users' approaches to children's oral health. Interest in early childhood oral health may also extend to other social media platforms, and it would be interesting to compare the use of Instagram to other applications. Greater details on users (i.e., who is posting on Instagram) would also be interesting for future research. It is important to note that the dataset (based on image posts, captions, and comments) relies on users' technological abilities and digital literacy to share what they want to share. Individuals without these skills may not be able to fully engage in what Instagram has to offer. It should also be noted that the search period for this study was up to July 2020, so more recent posts during COVID-19 pandemic times are missing. Regardless, this study provides interesting information on foundational early childhood oral health content on Instagram.

## Conclusion

Informational, instructional, promotional and positive Instagram graphics about children's oral health may provide increased oral health awareness, anticipatory guidance, and motivational support for the adoption of protective behaviours. Diverse posts could be appealing and contribute to oral hygiene awareness, earlier dental visits, and ECC prevention among various populations. There is a need to be deliberate in guiding social media content on preventive measures, and for responding to social media users who visit platforms seeking health information. Instagram is being used by many different types of people, and users are providing and consuming lots of content related to early childhood oral health. Users are and will continue to seek information about ECC. Oral health providers should consider Instagram as a platform to share simple key messages that promote early childhood oral health, and to educate parents and caregivers on how to prevent ECC. More useful and factual information that captures the attention of parents/caregivers is needed. Professional guidance should accompany the use of social platforms for the best results.

## Data Availability

The raw data supporting the conclusions of this article will be made available by the authors, without undue reservation.
